# Doctors and the Speed Limit

**Published:** 1910-07-09

**Authors:** 


### Doctors and the Speed Limit.

A good deal has been heard during the past twelvemonth of the justice and policy of allowing medical men, when travelling in motor-cars to see their patients, to exceed the particular speed limit legally in force in the district where it may happen to have been violated. The point arose originally when a well-known surgeon was prosecuted for this offence and excused himself upon the ground that he was hurrying to see an urgent case. This original defence so captivated the bench that the offender was let off. On a good many subsequent occasions the same defence has been put forward by medical motorists, with varying degrees of success. In the main, public opinion seems to have inclined towards allowing some licence to the doctor who can show that he is really travelling at speed to see a patient, though a good many people have been rather sceptical as to the urgency of many of the cases in which this has occurred; and, to speak frankly, it is practically certain that the tacit privilege has been a good many times abused by the profession.

A recent letter in the *Daily Mail* puts the gist of the matter from the lay standpoint with such common-sense that we feel constrained to draw the attention of medical men to it, notwithstanding that the writer of it would withhold altogether any licence or liberty to exceed the recognised limits. He asks those who would concede what some medical motorists ask for: Did anyone ever see a doctor not in a motor-car breaking speed records? And here he has laid his finger on one of the weak spots in the position he is attacking. He has, he says, never seen a doctor furiously lashing his horse or scorching on his bicycle in the endeavour to gain a few minutes or seconds in the race of life and death. "It is strange that as soon as he becomes the possessor of a motor-car the doctor gains a new sense of the urgency of his mission. He can travel twenty miles an hour where previously he could only go eight, but this does not satisfy him. He feels that considerations of humanity entitle him to get the very top speed out of his engine. The fact of the matter is, it is because he is a motorist, not because he is a doctor, that he wants to exceed the speed limit." This indictment, severe though it may appear, contains a half-truth which it is all the more difficult to rebut. To assert, as the *Daily Mail's* correspondent does, that a doctor is never known to hurry himself until he has a car is a libel on the profession and a most unjustified one. But, for all the exaggeration, the last sentence of the paragraph quoted above sums up very neatly a view of the question which many people have felt without being able to voice so articulately as has here been done.

But the thrust is pressed still further home by the Salisbury letter-writer. He proceeds to point out that if the doctor hurrying to the relief of his patient is entitled to rush along at forty miles an hour, surely the patient seeking the assistance of his doctor has the right to make the same speed. For it is not the doctor's right, but the patient's urgent need, that demands the excessive speed. If every patient on his way to the doctor's consulting-room is to be entitled to go as fast as he likes, it will be an unimaginative motorist who will not be able to provide himself with a convenient qualifying ailment. And, it may be added, it will prove the opening of an era of rare prosperity for doctors.

